# Tobacco Use Assessment and Cessation Medication Orders Among Primary Care Patients by Ethnicity, Sex, and Acculturation Indicators

**DOI:** 10.1007/s11606-025-09836-5

**Published:** 2025-10-01

**Authors:** Steffani R. Bailey, Jun Hwang, Jennifer A. Lucas, Kristin Lyon-Scott, Miguel Marino, Roopradha Datta, Ana R. Quiñones, Brian Chan, John Heintzman

**Affiliations:** 1 Department of Family Medicine, Oregon Health & Science University, Mailcode FM, 3181 SW Sam Jackson Park Rd, Portland, OR 97239 USA; 2 OCHIN, Inc, Portland, OR USA; 3 Department of Medicine, Addiction Medicine Section, Oregon Health & Science University, Portland, OR USA

**Keywords:** tobacco, ethnicity, primary care, language preference, nativity, acculturation

## Abstract

**Background:**

Lung cancer is a leading cause of cancer death among Hispanic men and women in the United States. Smoking rates vary among Hispanic subgroups, with higher rates among those with indicators (e.g., language preference, nativity) of greater acculturation. It is recommended that clinicians ask about tobacco use and provide cessation treatment, as warranted. While studies in health care settings note disparities in tobacco-related care by ethnicity and acculturation proxies, we are unaware of studies that have evaluated these relationships among adult patients within community-based health care clinics (CHCs), key settings for provision of care for Hispanic patients.

**Objective:**

To examine rates of tobacco use assessment and cessation medication orders among Hispanic patients by language and nativity compared to non-Hispanic White patients in CHCs.

**Design:**

Retrospective observational study using electronic health record (EHR) data.

**Patients:**

1,016,391 adult patients with ≥ 1 primary care visit to a study CHC between 9/1/2020–9/1/2022.

**Main Outcomes:**

Outcomes included tobacco use assessment and, among those identified as using tobacco, having a cessation medication ordered. The primary independent variable combined ethnicity and language preference, with sensitivity analyses combining ethnicity and nativity. We used separate generalized estimating equation regressions for each sex to estimate risk differences of each outcome by patient subgroups, adjusting for covariates.

**Key Results:**

Compared with non-Hispanic White patients, Spanish-preferring Hispanic males and females and English-preferring Hispanic males had higher rates of having tobacco use assessed (covariate-adjusted risk differences [aRD] = 2.23%, 95% CI = 1.78%—2.69%; 1.74%, 95% CI = 1.35%—2.14%; 0.41%, 95% CI = 0.12%—0.70%, respectively). Ethnicity/nativity analyses found higher rates of tobacco assessment among all Hispanic subgroups. Compared with non-Hispanic White patients, all Hispanic patient subgroups had lower rates of having a cessation medication ordered.

**Conclusions:**

Efforts are needed to inform, develop and test culturally-appropriate and patient-centered interventions for tobacco cessation among Hispanic patients.

**Supplementary Information:**

The online version contains supplementary material available at 10.1007/s11606-025-09836-5.

## INTRODUCTION

Over 80% of lung cancer cases in the United States are attributable to smoking.^1^ Among Hispanic individuals, lung cancer is the leading cause of cancer death in men and the second leading cause in women.^2^ Although smoking prevalence is lower among Hispanic adults compared with non-Hispanic adults^3^, greater acculturation is linked to higher rates of smoking among Hispanic persons,^4,5^ particularly among females. ^4,6–8^ Acculturation, commonly measured via proxies such as language preference and nativity,^9,10^ is often defined as a change in cultural identity that occurs as the result of contact between two or more cultural groups and the degree to which the culture of origin is maintained and the new or dominant culture is adopted.^11,12^ Literature suggests that many (but not all) Latin American countries have more negative views of smoking than in the US, especially among females;^13^ thus, as a person adopts US social norms and culture, which can be influenced by exposure to targeted tobacco marketing,^14^ they might be more likely to initiate smoking than those with lower acculturation.

The United States Preventive Services Task Force recommends that clinicians ask all adults about tobacco use, and among those who are currently using, advise them to quit and provide appropriate tobacco cessation treatment.^15^ Most studies find that Hispanic adults who smoke are less likely to be asked by a health care provider about tobacco use,^16,17^ to receive advice to quit,^18–21^ and to use evidence-based cessation treatments than non-Hispanic white persons who smoke,^19,20,22–25^ despite similar^19,20^ or even increased likelihood,^18^ of making a quit attempt. However, a study using data from 2015–2018 National Ambulatory Medical Care Surveys found no differences in receipt of counseling or medication provided in primary care settings between non-Hispanic White persons and those who were Hispanic.^26^ Consistent with other studies,^27,28^ females were more likely to receive medications than males but this was not examined by ethnicity.^26^

Studies have shown that proxies for acculturation among Hispanic adults are associated with varying health care access and utilization.^29–32^ We are unaware of any studies that have evaluated rates of tobacco use assessment and cessation assistance by ethnicity (Hispanic, non-Hispanic), sex (male, female) and acculturation indicators (language preference, nativity) among patients seen in community-based health care clinic (CHC) settings. While one study using CHC data from older adults found that, compared with non-Hispanic White patients, Spanish-preferring Hispanic patients had significantly lower odds of having a tobacco use assessment and both Spanish- and English-preferring Hispanic patients had lower odds of having a cessation medication order, this study was limited to older adults and did not examine differences by patient sex or nativity.^33^ We focus on CHCs, which are important settings for Hispanic patients to receive health care, as they comprise 37% of all CHC patients.^34^ Thus, these clinical settings are important entry points to ensure access to care and preventive services for Hispanic patients, and can have a significant impact on reducing known health care-related disparities.^35,36^

The current study used electronic health record (EHR) data from adult patients seen in primary care CHCs to examine whether rates of tobacco assessment and cessation medication orders vary by patient ethnicity and language preference (primary analysis), as well as ethnicity and nativity (sensitivity analyses), separately for males and females. Given the higher tobacco use among non-Hispanic White adults, we hypothesized lower rates of tobacco use assessment among all Hispanic patient subgroups compared with non-Hispanic White patients, with the largest differences between non-Hispanic White and both Spanish-preferring Hispanic patients and foreign-born Hispanic patients. We hypothesized similar relationships between these patient subgroups and cessation medication orders.

## METHODS

### Data Source

Electronic health record (EHR) data were extracted from clinics on the OCHIN network (N = 2,610 clinics from 28 states). OCHIN is a national nonprofit health IT consultancy that provides a fully hosted and shared EHR platform across every clinic in the network.^37^ The same discrete fields within the EHR are available across the OCHIN network of clinics, including those to document assessment of tobacco use and medication orders. This study was approved by our university Institutional Review Board.

### Study Population

We used EHR data from patients ≥ 18 years of age with ≥ 1 visit to a primary care CHC (n = 926 clinics) across 25 states between 9/1/2020–9/1/2022. We limited our population to those who were either non-Hispanic White, Spanish-preferring Hispanic or English-preferring Hispanic. Subsets of this study population were used to assess nativity among those who had it documented and/or for whom it could be predicted. CHC primary care clinics included federally qualified health centers, county clinics, not-for-profit clinics and rural health clinics with department specialties of primary care, family medicine, or internal medicine.

### Variables

#### Tobacco Use Assessment

The denominator included all patients who met the study criteria. The numerator included patients who were assessed for tobacco use (e.g., asked about tobacco use) at one or more encounters in the study period. Tobacco use is defined here as the use of a smoked or smokeless tobacco product. A patient was identified as having an assessment if the EHR record indicated a tobacco status (e.g., current, former, never) as opposed to unknown.

#### Current Tobacco Use

Among patients assessed for tobacco use, patients were defined as currently using tobacco if they were identified as using smoked or smokeless tobacco products at any point in the study period as recorded in the EHR.

#### Cessation Medication Ordered

Among patients identified as currently using tobacco, cessation medication orders (i.e., bupropion, varenicline, nicotine replacement products) were extracted from the medication orders in the EHR.

#### Independent Variables

The primary independent variable combined ethnicity and language preference (i.e., non-Hispanic White, Spanish-preferring Hispanic, English-preferring Hispanic), with patients stratified by sex (male, female). For sensitivity analyses, we created an independent variable that combined ethnicity and nativity in a similar fashion (i.e., non-Hispanic White, US-born Hispanic, foreign-born Hispanic) and defined it on two separate subsets of patients: one that included Hispanic patients with documented nativity and one that included both those with documented nativity and with nativity predicted via machine learning methods. Both language preference and nativity, if self-reported, are documented in the EHR.

#### Covariates

The following patient data were derived from visits within primary care clinics: age at first visit in the study period, number of visits over the study period, proportion of visits conducted via telehealth, medical conditions (hypertension [based on 140/90 threshold], heart disease, lung disease, diabetes, cancers including breast, cervical, colorectal, liver, lung, prostate, stomach), psychiatric conditions (depressive disorders, anxiety disorders) and substance use disorders, identified via the International Classification of Diseases, Ninth and 10th Revisions codes in the problem list or encounter diagnoses. Household income (percent of federal poverty level [FPL], categorized as ≤ 138% for ≥ 50% of visits, ≤ 138% for < 50% of visits, unknown), insurance (categorized here as never insured, some private never public, some public never private, mixture of private and public) and body mass index (BMI) data were not limited to data only from primary care clinics. Clinic state also was included as a covariate.

### Statistical Analysis

Descriptive statistics were conducted to examine patient characteristics. For each binary outcome (tobacco use assessment; cessation medication ordered) and within each sex stratum (male; female), we used a multivariable generalized estimating equation (GEE) regression model under the identity link, clustering by primary care clinic using an exchangeable working correlation structure to estimate risk differences (RDs) and their 95% confidence intervals (CI) comparing English-preferring Hispanic, and Spanish-preferring Hispanic to non-Hispanic White patients, adjusting for the covariates noted above. Analogous models were run for the sensitivity analyses using the independent variables combining ethnicity and nativity. Details of the machine learning model used to predict US- and foreign-born status for patients missing nativity data are provided in Appendix 1.

## RESULTS

### Study Population

Table 1 presents descriptive statistics for the overall study sample (N = 1,016,391 patients) and by ethnicity/preferred language/sex. Our sample consisted of 56.5% females, the majority of the overall sample were between 35–64 years of age, 53.8% had some public insurance with no indication of private insurance at any visit, 15% were not insured at any visit, and 69.3% had household incomes ≤ 138% of the FPL at most visits. See Appendix Tables 1 and 2 for study sample characteristics of those included in the nativity analyses.

**Table 1:** Patient Characteristics Overall and by Ethnicity/Preferred Language

	Overall	Non-Hispanic White Females	Spanish-preferring Hispanic Females	English-preferring Hispanic Females	Non-Hispanic White Males	Spanish-preferring Hispanic Males	English-preferring Hispanic Males
N (%)	1016391	247932	213057	113201	212885	143746	85570
Tobacco use assessed	812300 (79.9%)	199679 (80.5%)	182462 (85.6%)	87078 (76.9%)	167363 (78.6%)	113043 (78.6%)	62675 (73.2%)
Current use (among assessed)	165909 (20.4%)	58080 (29.1%)	6838 (3.7%)	10,495 (12.1%)	60888 (36.4%)	15169 (13.4%)	14439 (23.0%)
Medication*							
Any below	40382 (24.3%)	17324 (29.8%)	1008 (14.7%)	2131 (20.3%)	15560 (25.6%)	1923 (12.7%)	2436 (16.9%)
Bupropion	13581 (8.2%)	6481 (11.2%)	303 (4.4%)	712 (6.8%)	5033 (8.3%)	371 (2.4%)	681 (4.7%)
Nicotine	26545 (16.0%)	10640 (18.3%)	717 (10.5%)	1430 (13.6%)	10476 (17.2%)	1521 (10.0%)	1761 (12.2%)
Varenicline	8161 (4.9%)	3873 (6.7%)	164 (2.4%)	359 (3.4%)	3072 (5.0%)	320 (2.1%)	373 (2.6%)
Age in years							
18–20	59206 (5.8%)	11608 (4.7%)	7712 (3.6%)	15296 (13.5%)	7546 (3.5%)	6895 (4.8%)	10149 (11.9%)
21–24	67326 (6.6%)	15405 (6.2%)	6739 (3.2%)	17697 (15.6%)	10707 (5.0%)	5499 (3.8%)	11279 (13.2%)
25–34	194631 (19.1%)	49534 (20.0%)	27017 (12.7%)	34196 (30.2%)	41091 (19.3%)	18739 (13.0%)	24054 (28.1%)
35–49	307959 (30.3%)	63828 (25.7%)	86981 (40.8%)	27047 (23.9%)	54929 (25.8%)	53243 (37.0%)	21931 (25.6%)
50–64	271339 (26.7%)	69649 (28.1%)	62182 (29.2%)	14717 (13.0%)	66457 (31.2%)	43801 (30.5%)	14533 (17.0%)
65 +	115930 (11.4%)	37908 (15.3%)	22426 (10.5%)	4248 (3.8%)	32155 (15.1%)	15569 (10.8%)	3624 (4.2%)
Insurance							
Never	152193 (15.0%)	15255 (6.2%)	48417 (22.7%)	11314 (10.0%)	17245 (8.1%)	47571 (33.1%)	12391 (14.5%)
Some private	207371 (20.4%)	58149 (23.5%)	40676 (19.1%)	17543 (15.5%)	51503 (24.2%)	24645 (17.1%)	14855 (17.4%)
Some public	547261 (53.8%)	141028 (56.9%)	97785 (45.9%)	73602 (65.0%)	121588 (57.1%)	61130 (42.5%)	52128 (60.9%)
Private/public	108205 (10.6%)	33406 (13.5%)	25730 (12.1%)	10642 (9.4%)	22441 (10.5%)	9891 (6.9%)	6095 (7.1%)
Unknown	1361 (0.1%)	94 (0.0%)	449 (0.2%)	100 (0.1%)	108 (0.1%)	509 (0.4%)	101 (0.1%)
Visit prior to study period	510689 (50.2%)	137224 (55.3%)	112829 (53.0%)	50838 (44.9%)	110984 (52.1%)	64171 (44.6%)	34643 (40.5%)
Visits during study period							
≥ 1 to ≤ 3	385403 (37.9%)	81129 (32.7%)	69620 (32.7%)	47300 (41.8%)	81469 (38.3%)	63560 (44.2%)	42325 (49.5%)
> 3 to ≤ 5	122322 (12.0%)	27245 (11.0%)	24667 (11.6%)	14423 (12.7%)	25756 (12.1%)	18712 (13.0%)	11519 (13.5%)
> 5 to ≤ 10,	177758 (17.5%)	42894 (17.3%)	37730 (17.7%)	19903 (17.6%)	37906 (17.8%)	25047 (17.4%)	14278 (16.7%)
> 10	330908 (32.6%)	96664 (39.0%)	81040 (38.0%)	31575 (27.9%)	67754 (31.8%)	36427 (25.3%)	17448 (20.4%)
Fraction of telehealth visits^†^							
< 1/3	707324 (69.6%)	178095 (71.8%)	150342 (70.6%)	70194 (62.0%)	155113 (72.9%)	98830 (68.8%)	54750 (64.0%)
≥ 1/3- ≤ 2/3	194267 (19.1%)	43098 (17.4%)	43159 (20.3%)	26449 (23.4%)	35230 (16.5%)	28097 (19.5%)	18234 (21.3%)
> 2/3	114800 (11.3%)	26739 (10.8%)	19556 (9.2%)	16558 (14.6%)	22542 (10.6%)	16819 (11.7%)	12586 (14.7%)
FPL (by % visits)							
≤ 138 for ≥ 50%	704474 (69.3%)	150249 (60.6%)	176160 (82.7%)	82889 (73.2%)	125012 (58.7%)	110917 (77.2%)	59247 (69.2%)
≤ 138 for < 50%	185482 (18.2%)	58774 (23.7%)	23683 (11.1%)	15805 (14.0%)	52443 (24.6%)	20900 (14.5%)	13877 (16.2%)
Unknown	126435 (12.4%)	38909 (15.7%)	13214 (6.2%)	14507 (12.8%)	35430 (16.6%)	11929 (8.3%)	12446 (14.5%)
BMI							
Ever obese	396905 (39.1%)	92249 (37.2%)	101127 (47.5%)	48081 (42.5%)	69175 (32.5%)	54694 (38.0%)	31579 (36.9%)
Ever OW/never obese	242361 (23.8%)	48264 (19.5%)	57806 (27.1%)	21692 (19.2%)	53493 (25.1%)	42186 (29.3%)	18920 (22.1%)
Never obese/OW	156297 (15.4%)	52686 (21.3%)	22,256 (10.4%)	17058 (15.1%)	38999 (18.3%)	13,566 (9.4%)	11732 (13.7%)
Unknown	220828 (21.7%)	54733 (22.1%)	31,868 (15.0%)	26370 (23.3%)	51218 (24.1%)	33,300 (23.2%)	23339 (27.3%)
Hypertension^††^	314702 (31.0%)	74030 (29.9%)	62356 (29.3%)	21814 (19.3%)	78681 (37.0%)	52265 (36.4%)	25556 (29.9%)
Heart disease^§^	38330 (3.8%)	10562 (4.3%)	3413 (1.6%)	1450 (1.3%)	15942 (7.5%)	4574 (3.2%)	2389 (2.8%)
Lung disease^‖^	131198 (12.9%)	51813 (20.9%)	15751 (7.4%)	15201 (13.4%)	32493 (15.3%)	7315 (5.1%)	8625 (10.1%)
Diabetes	162328 (16.0%)	27226 (11.0%)	47015 (22.1%)	11547 (10.2%)	28559 (13.4%)	36414 (25.3%)	11567 (13.5%)
Depressive disorder	228129 (22.4%)	87530 (35.3%)	36937 (17.3%)	26852 (23.7%)	51,494 (24.2%)	11957 (8.3%)	13359 (15.6%)
Anxiety or PTSD	266629 (26.2%)	104393 (42.1%)	37445 (17.6%)	32227 (28.5%)	60646 (28.5%)	15067 (10.5%)	16851 (19.7%)
SUD other than tobacco	107211 (10.5%)	33689 (13.6%)	1816 (0.9%)	6966 (6.2%)	43711 (20.5%)	9175 (6.4%)	11854 (13.9%)
Any cancer^¶^	17843 (1.8%)	7298 (2.9%)	3278 (1.5%)	1028 (0.9%)	4290 (2.0%)	1387 (1.0%)	562 (0.7%)

^*^Tobacco cessation medication order among those reporting current tobacco use

^†^During study period of September 1, 2020 through September 1, 2022

^††^Any blood pressure readings ≥ 140/90 systolic/diastolic during the observation period

^§^Ischemic heart disease, or heart failure and non-ischemic heart disease

^‖^Chronic obstructive pulmonary disease, bronchiectasis, or asthma

^¶^Breast, cervical, colorectal, liver, lung, prostate, stomach

Notes. Cell sizes ≤ 10 have been masked to protect patients’ identities. To prevent back-calculating, we have masked an additional column cell

States where clinics are located: AK, AL, CA, CT, FL, GA, ID, IL, IN, LA, MA, MN, MT, NC, NJ, NM, NV, OH, OK, OR, SC, TX, UT, WA, WI

FPL: federal poverty level; OW: overweight; PTSD: Post-traumatic stress disorder; SUD: substance use disorder

### Tobacco Use Assessment

**Figure 1:**
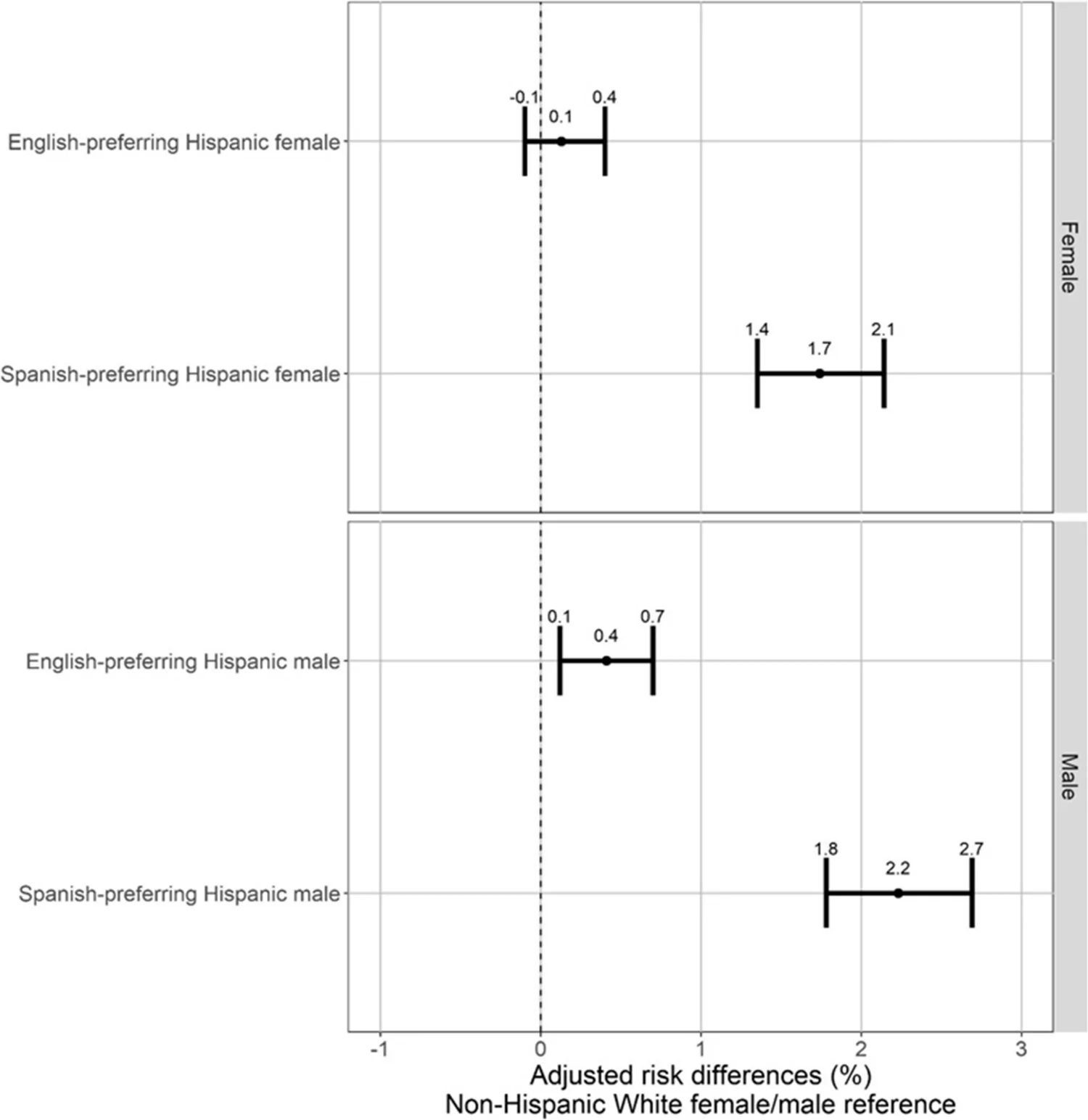
Adjusted risk differences (%) of tobacco use assessment comparing groups defined by ethnicity and language preference to non-Hispanic White patients (reference) (analysis sample size = 1,016,391 patients). Models were specified as generalized estimating equations under the identity link, clustering by primary care clinic using an exchangeable working correlation, stratified by sex, adjusting for age at first visit in the study period, number of visits over the study period, proportion of visits conducted via telehealth, medical conditions (hypertension, heart disease, lung disease, diabetes, cancers including breast, cervical, colorectal, liver, lung, prostate, stomach), psychiatric conditions (depressive disorders, anxiety disorders) and substance use disorders, household income (percent of federal poverty level), insurance category, body mass index, and state in which clinic is located.

Overall, 79.9% of all patients within our primary sample had tobacco use assessed within the 2-year study period. Assessment among female subgroups ranged from 76.9% (English-preferring Hispanic) to 85.6% (Spanish-preferring Hispanic) and from 73.2% (English-preferring Hispanic) to 78.6% (Spanish-preferring Hispanic) among male subgroups. In adjusted models, compared with non-Hispanic White patients, Spanish-preferring Hispanic patients had significantly higher rates of having tobacco use assessed (covariate-adjusted risk difference [aRD] = 2.23%, 95% CI = 1.78%—2.69% for males; 1.74%, 95% CI = 1.35%—2.14% for females), as did English-preferring males (aRD = 0.41%, 95% CI = 0.12%—0.70%). See Fig. 1. For both nativity models (e.g., documented, predicted) foreign and US-born Hispanic males and females had significantly higher estimated rates of tobacco use assessment compared with their non-Hispanic White counterparts. The risk differences were of similar magnitude for both nativity models and higher than those estimated using language preference. See Appendix Fig. 1.

### Current Tobacco Use

Of the 79.9% of patients who had tobacco use assessed, 20.4% were documented as currently using tobacco. Among men and women, non-Hispanic White patients had the highest tobacco use rates (36.4% male; 29.1% female) and Spanish-preferring Hispanic patients had the lowest rates (13.4% male; 3.7% female). See Table 1. For rates of tobacco use by nativity samples, see Appendix Tables 1 and 2.

### Cessation Medication Orders

**Figure 2:**
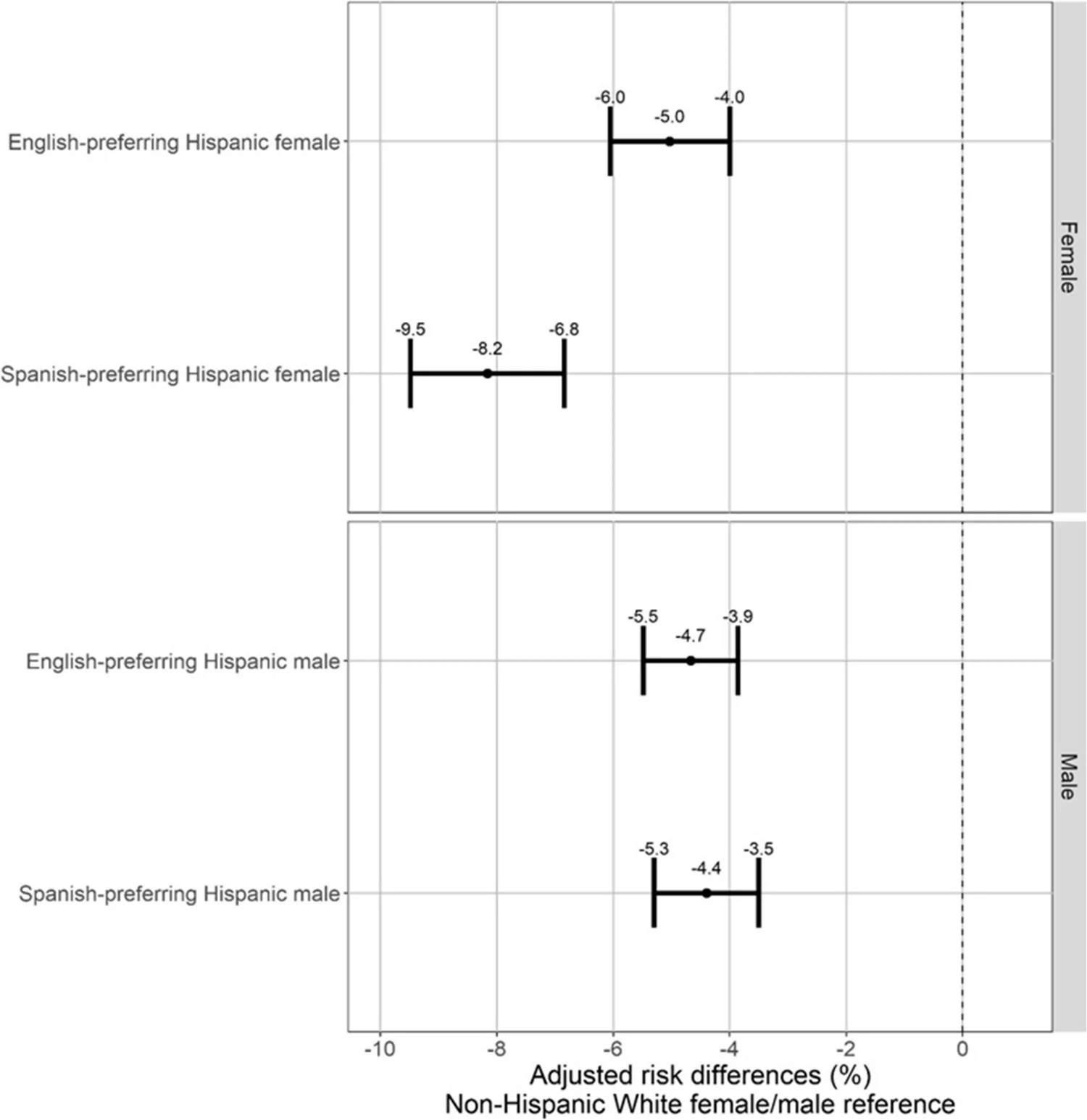
Adjusted risk differences (%) of cessation medication orders comparing groups defined by ethnicity and language preference to non-Hispanic White patients (reference), among patients identified as currently using tobacco during the study period (analysis sample size = 165,909 patients). Models were specified as generalized estimating equations under the identity link, clustering by primary care clinic using an exchangeable working correlation, stratified by sex, adjusting for age at first visit in the study period, number of visits over the study period, proportion of visits conducted via telehealth, medical conditions (hypertension, heart disease, lung disease, diabetes, cancers including breast, cervical, colorectal, liver, lung, prostate, stomach), psychiatric conditions (depressive disorders, anxiety disorders) and substance use disorders, household income (percent of federal poverty level), insurance category, body mass index, and state in which clinic is located.

Of patients identified as currently using tobacco during the study period, almost a quarter of patients had at least one order for a cessation medication, with nicotine replacement therapy most common (16%) and lower prevalence for bupropion (8.2%) and varenicline (4.9%). Among males and females, non-Hispanic White patients had the highest percentage of having any cessation orders (25.6% and 29.8%, respectively). Spanish-preferring Hispanic males and females had the lowest prevalence of cessation orders (12.7% and 14.7%, respectively). See Table 1. In our adjusted models, compared with non-Hispanic White patients, all Hispanic subgroups had lower rates of having a cessation medication ordered, with the lowest rate seen among Spanish-preferring Hispanic females (aRD = −8.16%, 95% CI = −9.48%—−6.84%). See Fig. 2. Similarly, in analyses using patients with documented nativity, all Hispanic subgroups had lower rates of having a medication ordered, with the lowest among foreign-born Hispanic adults. Risk differences estimated using documented and machine learning-predicted nativity were also statistically significant with similar magnitudes to those estimated using only documented nativity. See Appendix Fig. 2.

## DISCUSSION

Consistent with the literature, we found wide variation in tobacco use between subgroups of patients, with the highest prevalences among non-Hispanic White patients, and lowest among Hispanic patients with proxy indicators of lower acculturation (Spanish-preferring, foreign-born). Contrary to our hypothesis that non-Hispanic White patients would have the highest rates of tobacco use assessment given their substantially higher rates of tobacco use, those with lower acculturation (both Spanish-preferring and foreign-born) had higher rates of assessment compared with their non-Hispanic White counterparts. This is surprising given that these groups have the lowest prevalence of tobacco use of all subgroups and most studies have found that tobacco-related care is lower among Hispanic patients compared to non-Hispanic White patients.^16–21^ Further, this is contrary to what was found in a prior study limited to older adults in CHCs, where older Spanish-preferring Hispanic patients were *less likely* to have been assessed for tobacco use.^33^ Given that greater acculturation is associated with increased uptake of tobacco use,^38^ it is possible that clinicians are screening to prevent uptake or progression of tobacco use as one becomes more acculturated, and may be more likely to do so among younger patients who might be more at risk.

We did see some differences by proxy indicators of acculturation. In our nativity analyses, all Hispanic subgroups had higher rates of tobacco use assessment than non-Hispanic White patients. In our primary analyses focused on language preference, there was not a statistically significant difference in tobacco use assessment between US-born Hispanic females and non-Hispanic White females. Further, the magnitudes of all estimates were larger for nativity compared to preferred language. These differences are not entirely surprising given that while both are indicators of acculturation, they are not identical. Further work is needed to expand the collection/availability of these data in order to give the clearest picture of provision of tobacco-related care for Latino patients in health care settings.

As hypothesized, among patients identified as currently using tobacco, we found significantly lower rates of having a cessation medication ordered among all Hispanic patients, with the lowest among those with lower acculturation (Spanish-preferring, foreign-born). These findings are consistent with the majority of published literature ^19,20,22–25^, although to our knowledge, this paper is one of the first to examine these relationships within CHC settings and to include both language and nativity variables.

A 2017 review of potential reasons for disparities in cessation medication use among Hispanic patients identified avoidance of medications in general, misconceptions or distrust of tobacco cessation medications, and perceptions of smoking as a weakness that can be overcome by will-power, rather than a medical condition treated with pharmacotherapy.^25^ However, an analysis of the 2022 National Health Information Survey that found Hispanic patients who had seen a health care provider within the past 12 months were less likely to be advised to quit compared to non-Hispanic White respondents,^21^ suggesting that treatment options might not even be offered.

Hispanic adults, as a whole, smoke less than non-Hispanic White adults,^39,40^ with Spanish-preferring patients smoking the least number of cigarettes per day.^41^ Despite the health effects of even light or intermediate cigarette smoking,^42^ clinicians might be less likely to provide advice to quit or to order cessation medications for patients who do not report a certain level of smoking. While we did not have data on smoking quantity/frequency to test this hypothesis, in a survey-based study of patients seen in an integrated health care delivery system, among those who reported smoking on some days or less than five cigarettes each day, English-speaking patients were more likely to report use of a cessation medication than Spanish-speaking patients, suggesting that smoking quantity is not, in fact, driving these differences.^43^

Few studies have tested interventions to support the uptake of pharmacotherapy among Hispanic patients. A recent randomized trial of over 450 English- and Spanish-preferring Hispanic adults compared standard care (printed educational material, access to free NRT via a toll-free study number) to a culturally accommodated mHealth cessation intervention consisting of standard care plus a 24-week text messaging counseling program. In addition to counseling, the text messages included prompts for accessing, using, and supporting pharmacotherapy. Compared to the standard care group, those in the mHealth intervention had higher uptake of NRT, as well as higher short-term abstinence rates.^44^ This is promising as over 70% of participants used the mHealth intervention in Spanish. Continued efforts are needed to develop and test culturally-appropriate and patient-centered interventions, including those that provide access and support for evidence-based pharmacotherapy for smoking cessation.

Our study has limitations. We do not have data on whether cessation medication orders were filled. We also do not have data available to determine whether a medication was not ordered because the clinician did not offer it or because the patient declined. There are limitations related to our use of EHR data, included missing data on patient nativity, potential inconsistencies in EHR documentation, and the inability to robustly assess whether advice to quit or more intensive cessation counseling was provided or if referrals were made for cessation assistance outside of the primary care setting (e.g., state Tobacco Quitlines). Our data spans across the time of the COVID-19 pandemic public health emergency (PHE); thus, we do not know if pre- or post-PHE rates would be similar. We do include a covariate for telehealth, as rates of tobacco assessment has been shown to be higher during in-person vs telehealth visits.^45,46^ Future studies are needed to examine whether rates of visit modalities (in-person, telephone, video) vary by ethnicity and indicators of acculturation in these CHC settings and if this impacted tobacco-related care differently for our subgroups of interest. We also do not have data to examine these relationships by specific county of birth for those born outside of the US. Finally, our study design does not allow us to assume any causal inference.

## CONCLUSIONS

We found consistently lower rates of having an order for tobacco cessation medications among all Hispanic subgroups compared with non-Hispanic White patients. Future research is needed to understand the etiology of these disparities to further inform the development of acceptable, appropriate, and targeted interventions to attain public health goals of improving health outcomes and wellbeing among Hispanic adults who use tobacco.

## Supplementary Information

Below is the link to the electronic supplementary material.

Supplementary Material 1 (DOCX 388 KB)

## Data Availability

Raw data underlying this article were generated from multiple health systems across the OCHIN network; restrictions apply to the availability and re-release of data under organizational agreements.
