# Supplementary material for: Tobacco Use Assessment and Cessation Medication Orders Among Primary Care Patients by Ethnicity, Sex, and Acculturation Indicators
Source: J Gen Intern Med. 2025 Oct 1;41(11):3121–9. doi: 10.1007/s11606-025-09836-5 (PMC13493682; doi:10.1007/s11606-025-09836-5)
Supplement: Supplementary file 1 [file 11606_2025_9836_MOESM1_ESM.docx]

**Sensitivity Analyses Methods**

The sensitivity analyses for this study comprise additional descriptive summaries and adjusted models utilizing alternative independent variables combining ethnicity and nativity of patients, i.e., non-Hispanic White (reference), US-born Hispanic, foreign-born Hispanic. The additional adjusted models are otherwise specified as the models are for the primary analysis, utilizing generalized estimating equations with the identity link under an exchangeable correlation structure and clustering by clinic, with the same dependent variables and covariates, and stratified by sex.

The first set of sensitivity analyses subsets Hispanic patients to those with documented nativity while the other also includes Hispanic patients for whom nativity could be predicted using a machine learning (ML) model, described after the results below. Hispanic patients with predicted nativity are categorized for descriptive summaries using the more probable predicted nativity value. For example, a Hispanic patient with a predicted probability of 0.85 of being US-born (and thus 0.15 of being foreign-born) would be descriptively categorized as US-born. For Hispanic patients with predicted nativity, the predicted probabilities of being US-born and foreign-born are used in lieu of binary values for those variables in the adjusted models.

**Machine Learning Approach**

Our approach is adapted from prior work by Marino et al.^1^ that predicted nativity based on electronic health records (EHR), neighborhood characteristics, and surname data. While the prior work was trained/validated on pediatric Hispanic patients, the predicted nativity values utilized in these sensitivity analyses were obtained from a model trained/validated on a larger sample of 1,500,191 Hispanic patients including adults, from 1,876 community health centers across 26 states (AK, AL, CA, CO, CT, FL, GA, ID, IL, IN, MA, MD, MN, MO, MT, NC, NJ, NM, NV, OH, OK, OR, TX, UT, WA, WI). This ML model exhibited excellent performance predicting US-born versus foreign-born nativity (area under the curve (AUC) = 0.90 on the withheld validation data set using 20% of the sample). A manuscript detailing the updated sample and performance for additional nativity outcomes is under preparation, while further details regarding the machine learning framework including predictors can be found in Marino et al.^1^

**REFERENCES**

1.	Marino M, Fankhauser K, Minnier J, et al. Disaggregating Latino nativity in equity research using electronic health records. *Health Services Research*. 2023;58:1119-1130. doi:10.1111/1475-6773.14154

| Appendix Table 1. Patient Characteristics Overall and by Ethnicity/Nativity for Subset with Documented Nativity | | | | | | |  |
| --- | --- | --- | --- | --- | --- | --- | --- |
|  | Overall | Non-Hispanic White Women | Foreign-born Hispanic Women | US-born Hispanic Women | Non-Hispanic White Men | Foreign-born Hispanic Men | US-born Hispanic Men |
| N (%) | 524986 | 247932 | 30416 | 9090 | 212885 | 18242 | 6421 |
| Tobacco use assessed | 419703 (79.9%) | 199679 (80.5%) | 26109 (85.8%) | 7393 (81.3%) | 167363 (78.6%) | 14367 (78.8%) | 4792 (74.6%) |
| Current use  (among assessed) | 123864 (29.5%) | 58080 (29.1%) | 928 (3.6%) | 1038 (14.0%) | 60888 (36.4%) | 1736 (12.1%) | 1194 (24.9%) |
| Medication* |  |  |  |  |  |  |  |
| Any below | 33721 (27.2%) | 17324 (29.8%) | 141 (15.2%) | 248 (23.9%) | 15560 (25.6%) | 210 (12.1%) | 238 (19.9%) |
| Bupropion | 11739 (9.5%) | 6481 (11.2%) | 41 (4.4%) | 78 (7.5%) | 5033 (8.3%) | 45 (2.6%) | 61 (5.1%) |
| Nicotine | 21759 (17.6%) | 10640 (18.3%) | 107 (11.5%) | 181 (17.4%) | 10476 (17.2%) | 170 (9.8%) | 185 (15.5%) |
| Varenicline | 7082 (5.7%) | 3873 (6.7%) | 24 (2.6%) | 47 (4.5%) | 3072 (5.0%) | 19 (1.1%) | 47 (3.9%) |
| Age in years |  |  |  |  |  |  |  |
| 18-20 | 22921 (4.4%) | 11608 (4.7%) | 845 (2.8%) | 1362 (15.0%) | 7546 (3.5%) | 638 (3.5%) | 922 (14.4%) |
| 21-24 | 30284 (5.8%) | 15405 (6.2%) | 1203 (4.0%) | 1277 (14.0%) | 10707 (5.0%) | 852 (4.7%) | 840 (13.1%) |
| 25-34 | 101951 (19.4%) | 49534 (20.0%) | 4541 (14.9%) | 2500 (27.5%) | 41091 (19.3%) | 2663 (14.6%) | 1622 (25.3%) |
| 35-49 | 140660 (26.8%) | 63828 (25.7%) | 11779 (38.7%) | 2089 (23.0%) | 54929 (25.8%) | 6537 (35.8%) | 1498 (23.3%) |
| 50-64 | 153244 (29.2%) | 69649 (28.1%) | 8898 (29.3%) | 1409 (15.5%) | 66457 (31.2%) | 5632 (30.9%) | 1199 (18.7%) |
| 65+ | 75926 (14.5%) | 37908 (15.3%) | 3150 (10.4%) | 453 (5.0%) | 32155 (15.1%) | 1920 (10.5%) | 340 (5.3%) |
| Insurance |  |  |  |  |  |  |  |
| Never | 42745 (8.1%) | 15255 (6.2%) | 4842 (15.9%) | >600 (>6%) | 17245 (8.1%) | 4235 (23.2%) | >500 (>8%) |
| Some private | 116183 (22.1%) | 58149 (23.5%) | 2995 (9.8%) | 922 (10.1%) | 51503 (24.2%) | 1887 (10.3%) | 727 (11.3%) |
| Some public | 300142 (57.2%) | 141028 (56.9%) | 16976 (55.8%) | 6226 (68.5%) | 121588 (57.1%) | 9841 (53.9%) | 4483 (69.8%) |
| Private/public | 65525 (12.5%) | 33406 (13.5%) | 5538 (18.2%) | 1318 (14.5%) | 22441 (10.5%) | 2173 (11.9%) | 649 (10.1%) |
| Unknown | 391 (0.1%) | 94 (<0.1%) | 65 (0.2%) | <11 (<1%) | 108 (0.1%) | 106 (0.6%) | <11 (<1%) |
| Visit prior to study period | 290474 (55.3%) | 137224 (55.3%) | 22016 (72.4%) | 5597 (61.6%) | 110984 (52.1%) | 11302 (62.0%) | 3351 (52.2%) |
| Visits during study period |  |  |  |  |  |  |  |
| ≥1 to ≤3 | 181728 (34.6%) | 81129 (32.7%) | 6965 (22.9%) | 2902 (31.9%) | 81469 (38.3%) | 6404 (35.1%) | 2859 (44.5%) |
| >3 to ≤5 | 58916 (11.2%) | 27245 (11.0%) | 2310 (7.6%) | 927 (10.2%) | 25756 (12.1%) | 1941 (10.6%) | 737 (11.5%) |
| >5 to ≤10, | 90690 (17.3%) | 42894 (17.3%) | 4307 (14.2%) | 1577 (17.3%) | 37906 (17.8%) | 2951 (16.2%) | 1055 (16.4%) |
| >10 | 193652 (36.9%) | 96664 (39.0%) | 16834 (55.3%) | 3684 (40.5%) | 67754 (31.8%) | 6946 (38.1%) | 1770 (27.6%) |
| Fraction of telehealth visits^†^ |  |  |  |  |  |  |  |
| <1/3 | 380221 (72.4%) | 178095 (71.8%) | 23512 (77.3%) | 6249 (68.7%) | 155113 (72.9%) | 13104 (71.8%) | 4148 (64.6%) |
| ≥1/3- ≤2/3 | 90019 (17.1%) | 43098 (17.4%) | 5007 (16.5%) | 1907 (21.0%) | 35230 (16.5%) | 3367 (18.5%) | 1410 (22.0%) |
| >2/3 | 54746 (10.4%) | 26739 (10.8%) | 1897 (6.2%) | 934 (10.3%) | 22542 (10.6%) | 1771 (9.7%) | 863 (13.4%) |
| FPL (by % visits) |  |  |  |  |  |  |  |
| ≤138 for ≥50% | 326551 (62.2%) | 150249 (60.6%) | 26219 (86.2%) | 6076 (66.8%) | 125012 (58.7%) | 14805 (81.2%) | 4190 (65.3%) |
| ≤138 for <50% | 120254 (22.9%) | 58774 (23.7%) | 3295 (10.8%) | 1770 (19.5%) | 52443 (24.6%) | 2633 (14.4%) | 1339 (20.9%) |
| Unknown | 78181 (14.9%) | 38909 (15.7%) | 902 (3.0%) | 1244 (13.7%) | 35430 (16.6%) | 804 (4.4%) | 892 (13.9%) |
| BMI |  |  |  |  |  |  |  |
| Ever obese | 188090 (35.8%) | 92249 (37.2%) | 13879 (45.6%) | 3937 (43.3%) | 69175 (32.5%) | 6511 (35.7%) | 2339 (36.4%) |
| Ever OW/never obese | 119027 (22.7%) | 48264 (19.5%) | 8457 (27.8%) | 1912 (21.0%) | 53493 (25.1%) | 5513 (30.2%) | 1388 (21.6%) |
| Never obese/OW | 98983 (18.9%) | 52686 (21.3%) | 3206 (10.5%) | 1405 (15.5%) | 38999 (18.3%) | 1772 (9.7%) | 915 (14.3%) |
| Unknown | 118886 (22.6%) | 54733 (22.1%) | 4874 (16.0%) | 1836 (20.2%) | 51218 (24.1%) | 4446 (24.4%) | 1779 (27.7%) |
| Hypertension^††^ | 170789 (32.5%) | 74030 (29.9%) | 8450 (27.8%) | 1890 (20.8%) | 78681 (37.0%) | 5963 (32.7%) | 1775 (27.6%) |
| Heart disease^§^ | 27677 (5.3%) | 10562 (4.3%) | 411 (1.4%) | 142 (1.6%) | 15942 (7.5%) | 456 (2.5%) | 164 (2.6%) |
| Lung disease^‖^ | 89131 (17.0%) | 51813 (20.9%) | 1962 (6.5%) | 1398 (15.4%) | 32493 (15.3%) | 727 (4.0%) | 738 (11.5%) |
| Diabetes | 68467 (13.0%) | 27226 (11.0%) | 6197 (20.4%) | 1110 (12.2%) | 28559 (13.4%) | 4443 (24.4%) | 932 (14.5%) |
| Depressive disorder | 150070 (28.6%) | 87530 (35.3%) | 5695 (18.7%) | 2517 (27.7%) | 51494 (24.2%) | 1648 (9.0%) | 1186 (18.5%) |
| Anxiety or PTSD | 176235 (33.6%) | 104393 (42.1%) | 5335 (17.5%) | 2712 (29.8%) | 60646 (28.5%) | 1794 (9.8%) | 1355 (21.1%) |
| SUD other than tobacco | 80431 (15.3%) | 33689 (13.6%) | 328 (1.1%) | 590 (6.5%) | 43711 (20.5%) | 1060 (5.8%) | 1053 (16.4%) |
| Any cancer^¶^ | 12380 (2.4%) | 7298 (2.9%) | 464 (1.5%) | 91 (1.0%) | 4290 (2.0%) | 193 (1.1%) | 44 (0.7%) |
| ^*^Tobacco cessation medication order among those reporting current tobacco use | | | | |  |  |  |
| ^†^During study period of September 1, 2020 through September 1, 2022 | | | |  |  |  |  |
| ^††^Any blood pressure readings ≥ 140/90 systolic/diastolic during the observation period | | | | |  |  |  |
| ^§^Ischemic heart disease, or heart failure and non-ischemic heart disease | | | |  |  |  |  |
| ^‖^Chronic obstructive pulmonary disease, bronchiectasis, or asthma | | | |  |  |  |  |
| ^¶^Breast, cervical, colorectal, liver, lung, prostate, stomach | | | |  |  |  |  |
| Notes. Cell sizes ≤10 have been masked to protect patients’ identities. To prevent back-calculating, we have masked an additional column cell. | | | | | | | |
| States where clinics are located: AK, AL, CA, CT, FL, GA, ID, IL, IN, MA, MN, MT, NC, NJ, NM, NV, OH, OK, OR, SC, TX, UT, WA, WI | | | | | | | |
| FPL: federal poverty level; OW: overweight; PTSD: Post-traumatic stress disorder; SUD: substance use disorder | | | | | | |  |

| Appendix Table 2. Patient Characteristics Overall and by Ethnicity/Nativity with Documented or Machine Learning-Predicted Nativity | | | | | | | |
| --- | --- | --- | --- | --- | --- | --- | --- |
|  | Overall | Non-Hispanic White Women | Foreign-born Hispanic Women | US-born Hispanic Women | Non-Hispanic White Men | Foreign-born Hispanic Men | US-born Hispanic Men |
| N (%) | 826076 | 247932 | 154689 | 67039 | 212885 | 96125 | 47406 |
| Tobacco use assessed | 686715 (83.1%) | 199679 (80.5%) | 139976 (90.5%) | 57390 (85.6%) | 167363 (78.6%) | 82874 (86.2%) | 39433 (83.2%) |
| Current use  (among assessed) | 151560 (22.1%) | 58080 (29.1%) | 4604 (3.3%) | 7737 (13.5%) | 60888 (36.4%) | 9954 (12.0%) | 10297 (26.1%) |
| Medication* |  |  |  |  |  |  |  |
| Any below | 38671 (25.5%) | 17324 (29.8%) | 694 (15.1%) | 1783 (23.0%) | 15560 (25.6%) | 1328 (13.3%) | 1982 (19.2%) |
| Bupropion | 13115 (8.7%) | 6481 (11.2%) | 211 (4.6%) | 575 (7.4%) | 5033 (8.3%) | 263 (2.6%) | 552 (5.4%) |
| Nicotine | 25322 (16.7%) | 10640 (18.3%) | 504 (10.9%) | 1221 (15.8%) | 10476 (17.2%) | 1041 (10.5%) | 1440 (14.0%) |
| Varenicline | 7941 (5.2%) | 3873 (6.7%) | 105 (2.3%) | 328 (4.2%) | 3072 (5.0%) | 227 (2.3%) | 336 (3.3%) |
| Age in years |  |  |  |  |  |  |  |
| 18-20 | 45482 (5.5%) | 11608 (4.7%) | 4373 (2.8%) | 11070 (16.5%) | 7546 (3.5%) | 3606 (3.8%) | 7279 (15.4%) |
| 21-24 | 50585 (6.1%) | 15405 (6.2%) | 4115 (2.7%) | 10953 (16.3%) | 10707 (5.0%) | 2822 (2.9%) | 6583 (13.9%) |
| 25-34 | 154274 (18.7%) | 49534 (20.0%) | 18890 (12.2%) | 20601 (30.7%) | 41091 (19.3%) | 10800 (11.2%) | 13358 (28.2%) |
| 35-49 | 245248 (29.7%) | 63828 (25.7%) | 64034 (41.4%) | 15431 (23.0%) | 54929 (25.8%) | 35243 (36.7%) | 11783 (24.9%) |
| 50-64 | 228772 (27.7%) | 69649 (28.1%) | 46353 (30.0%) | 7363 (11.0%) | 66457 (31.2%) | 31904 (33.2%) | 7046 (14.9%) |
| 65+ | 101715 (12.3%) | 37908 (15.3%) | 16924 (10.9%) | 1621 (2.4%) | 32155 (15.1%) | 11750 (12.2%) | 1357 (2.9%) |
| Insurance |  |  |  |  |  |  |  |
| Never | 95126 (11.5%) | 15255 (6.2%) | 28274 (18.3%) | 3165 (4.7%) | 17245 (8.1%) | 27791 (28.9%) | 3396 (7.2%) |
| Some private | 168995 (20.5%) | 58149 (23.5%) | 29605 (19.1%) | 7286 (10.9%) | 51503 (24.2%) | 16372 (17.0%) | 6080 (12.8%) |
| Some public | 459100 (55.6%) | 141028 (56.9%) | 72104 (46.6%) | 48422 (72.2%) | 121588 (57.1%) | 42560 (44.3%) | 33398 (70.5%) |
| Private/public | 102429 (12.4%) | 33406 (13.5%) | 24627 (15.9%) | 8153 (12.2%) | 22441 (10.5%) | 9282 (9.7%) | 4520 (9.5%) |
| Unknown | 426 (0.1%) | 94 (<0.1%) | 79 (0.1%) | 13 (<0.1%) | 108 (0.1%) | 120 (0.1%) | 12 (<0.1%) |
| Visit prior to study period | 490411 (59.4%) | 137224 (55.3%) | 109171 (70.6%) | 42990 (64.1%) | 110984 (52.1%) | 61651 (64.1%) | 28391 (59.9%) |
| Visits during study period |  |  |  |  |  |  |  |
| ≥1 to ≤3 | 251633 (30.5%) | 81129 (32.7%) | 28940 (18.7%) | 17554 (26.2%) | 81469 (38.3%) | 26480 (27.5%) | 16061 (33.9%) |
| >3 to ≤5 | 97783 (11.8%) | 27245 (11.0%) | 16348 (10.6%) | 8554 (12.8%) | 25756 (12.1%) | 12907 (13.4%) | 6973 (14.7%) |
| >5 to ≤10, | 157155 (19.0%) | 42894 (17.3%) | 31006 (20.0%) | 14118 (21.1%) | 37906 (17.8%) | 21148 (22.0%) | 10083 (21.3%) |
| >10 | 319505 (38.7%) | 96664 (39.0%) | 78395 (50.7%) | 26813 (40.0%) | 67754 (31.8%) | 35590 (37.0%) | 14289 (30.1%) |
| Fraction of telehealth visits^†^ |  |  |  |  |  |  |  |
| <1/3 | 587042 (71.1%) | 178095 (71.8%) | 111484 (72.1%) | 43287 (64.6%) | 155113 (72.9%) | 67777 (70.5%) | 31286 (66.0%) |
| ≥1/3- ≤2/3 | 161146 (19.5%) | 43098 (17.4%) | 33016 (21.3%) | 17192 (25.6%) | 35230 (16.5%) | 21077 (21.9%) | 11533 (24.3%) |
| >2/3 | 77888 (9.4%) | 26739 (10.8%) | 10189 (6.6%) | 6560 (9.8%) | 22542 (10.6%) | 7271 (7.6%) | 4587 (9.7%) |
| FPL (by % visits) |  |  |  |  |  |  |  |
| ≤138 for ≥50% | 568742 (68.8%) | 150249 (60.6%) | 131057 (84.7%) | 51319 (76.6%) | 125012 (58.7%) | 76523 (79.6%) | 34582 (72.9%) |
| ≤138 for <50% | 160136 (19.4%) | 58774 (23.7%) | 17649 (11.4%) | 9150 (13.6%) | 52443 (24.6%) | 14684 (15.3%) | 7436 (15.7%) |
| Unknown | 97198 (11.8%) | 38909 (15.7%) | 5983 (3.9%) | 6570 (9.8%) | 35430 (16.6%) | 4918 (5.1%) | 5388 (11.4%) |
| BMI |  |  |  |  |  |  |  |
| Ever obese | 331319 (40.1%) | 92249 (37.2%) | 78210 (50.6%) | 31348 (46.8%) | 69175 (32.5%) | 40764 (42.4%) | 19573 (41.3%) |
| Ever OW/never obese | 200590 (24.3%) | 48264 (19.5%) | 43543 (28.1%) | 13774 (20.5%) | 53493 (25.1%) | 30324 (31.5%) | 11192 (23.6%) |
| Never obese/OW | 135122 (16.4%) | 52686 (21.3%) | 15671 (10.1%) | 11229 (16.7%) | 38999 (18.3%) | 8941 (9.3%) | 7596 (16.0%) |
| Unknown | 159045 (19.3%) | 54733 (22.1%) | 17265 (11.2%) | 10688 (15.9%) | 51218 (24.1%) | 16096 (16.7%) | 9045 (19.1%) |
| Hypertension^††^ | 271071 (32.8%) | 74030 (29.9%) | 50022 (32.3%) | 13561 (20.2%) | 78681 (37.0%) | 39466 (41.1%) | 15311 (32.3%) |
| Heart disease^§^ | 35225 (4.3%) | 10562 (4.3%) | 2753 (1.8%) | 892 (1.3%) | 15942 (7.5%) | 3730 (3.9%) | 1346 (2.8%) |
| Lung disease^‖^ | 119291 (14.4%) | 51813 (20.9%) | 12526 (8.1%) | 10771 (16.1%) | 32493 (15.3%) | 5549 (5.8%) | 6139 (12.9%) |
| Diabetes | 134001 (16.2%) | 27226 (11.0%) | 36787 (23.8%) | 6917 (10.3%) | 28559 (13.4%) | 27928 (29.1%) | 6584 (13.9%) |
| Depressive disorder | 206406 (25.0%) | 87530 (35.3%) | 29677 (19.2%) | 19004 (28.3%) | 51494 (24.2%) | 9324 (9.7%) | 9377 (19.8%) |
| Anxiety or PTSD | 239330 (29.0%) | 104393 (42.1%) | 29413 (19.0%) | 22047 (32.9%) | 60646 (28.5%) | 11313 (11.8%) | 11518 (24.3%) |
| SUD other than tobacco | 98974 (12.0%) | 33689 (13.6%) | 1485 (1.0%) | 4990 (7.4%) | 43711 (20.5%) | 6804 (7.1%) | 8295 (17.5%) |
| Any cancer^¶^ | 16208 (2.0%) | 7298 (2.9%) | 2593 (1.7%) | 575 (0.9%) | 4290 (2.0%) | 1144 (1.2%) | 308 (0.6%) |
| ^*^Tobacco cessation medication order among those reporting current tobacco use | | | | |  |  |  |
| ^†^During study period of September 1, 2020 through September 1, 2022 | | | |  |  |  |  |
| ^††^Any blood pressure readings ≥ 140/90 systolic/diastolic during the observation period | | | | |  |  |  |
| ^§^Ischemic heart disease, or heart failure and non-ischemic heart disease | | | |  |  |  |  |
| ^‖^Chronic obstructive pulmonary disease, bronchiectasis, or asthma | | | |  |  |  |  |
| ^¶^Breast, cervical, colorectal, liver, lung, prostate, stomach | | | |  |  |  |  |
| States where clinics are located: AK, AL, CA, CT, FL, GA, ID, IL, IN, LA, MA, MN, MT, NC, NJ, NM, NV, OH, OK, OR, SC, TX, UT, WA, WI | | | | | | | |
| FPL: federal poverty level; OW: overweight; PTSD: Post-traumatic stress disorder; SUD: substance use disorder | | | | | | |  |

**Appendix Figure 1**. Adjusted risk differences (%) of tobacco use assessment comparing groups defined by ethnicity and nativity to non-Hispanic White patients (reference). Panel A: Hispanic patients subsetted with documented nativity (n = 524,986). Panel B. Hispanic patients subsetted with documented nativity or machine learning-predicted nativity (n = 826,076). Models were specified as generalized estimating equations under the identity link, clustering by primary care clinic using an exchangeable working correlation, stratified by sex, adjusting for age at first visit in the study period, number of visits over the study period, proportion of visits conducted via telehealth, medical conditions (hypertension, heart disease, lung disease, diabetes, cancers including breast, cervical, colorectal, liver, lung, prostate, stomach), psychiatric conditions (depressive disorders, anxiety disorders) and substance use disorders, household income (percent of federal poverty level), insurance category, body mass index, and state in which clinic is located.

**Appendix Figure 2**. Adjusted risk differences (%) of cessation medication orders comparing groups defined by ethnicity and language preference to non-Hispanic White patients (reference), among patients identified as currently using tobacco during the study period. Panel A: Hispanic patients subsetted with documented nativity (n = 123,864). Panel B. Hispanic patients subsetted with documented nativity or machine learning-predicted nativity (n = 151,560). Models were specified as generalized estimating equations under the identity link, clustering by primary care clinic using an exchangeable working correlation, stratified by sex, adjusting for age at first visit in the study period, number of visits over the study period, proportion of visits conducted via telehealth, medical conditions (hypertension, heart disease, lung disease, diabetes, cancers including breast, cervical, colorectal, liver, lung, prostate, stomach), psychiatric conditions (depressive disorders, anxiety disorders) and substance use disorders, household income (percent of federal poverty level), insurance category, body mass index, and state in which clinic is located.
